# Supplementary figures and images for: Environmental and Nutritional Factors That Affect Growth and Metabolism of the Pneumococcal Serotype 2 Strain D39 and Its Nonencapsulated Derivative Strain R6
Source: PLoS One. 2013 Mar 7;8(3):e58492. doi: 10.1371/journal.pone.0058492 (PMC3591343; doi:10.1371/journal.pone.0058492)

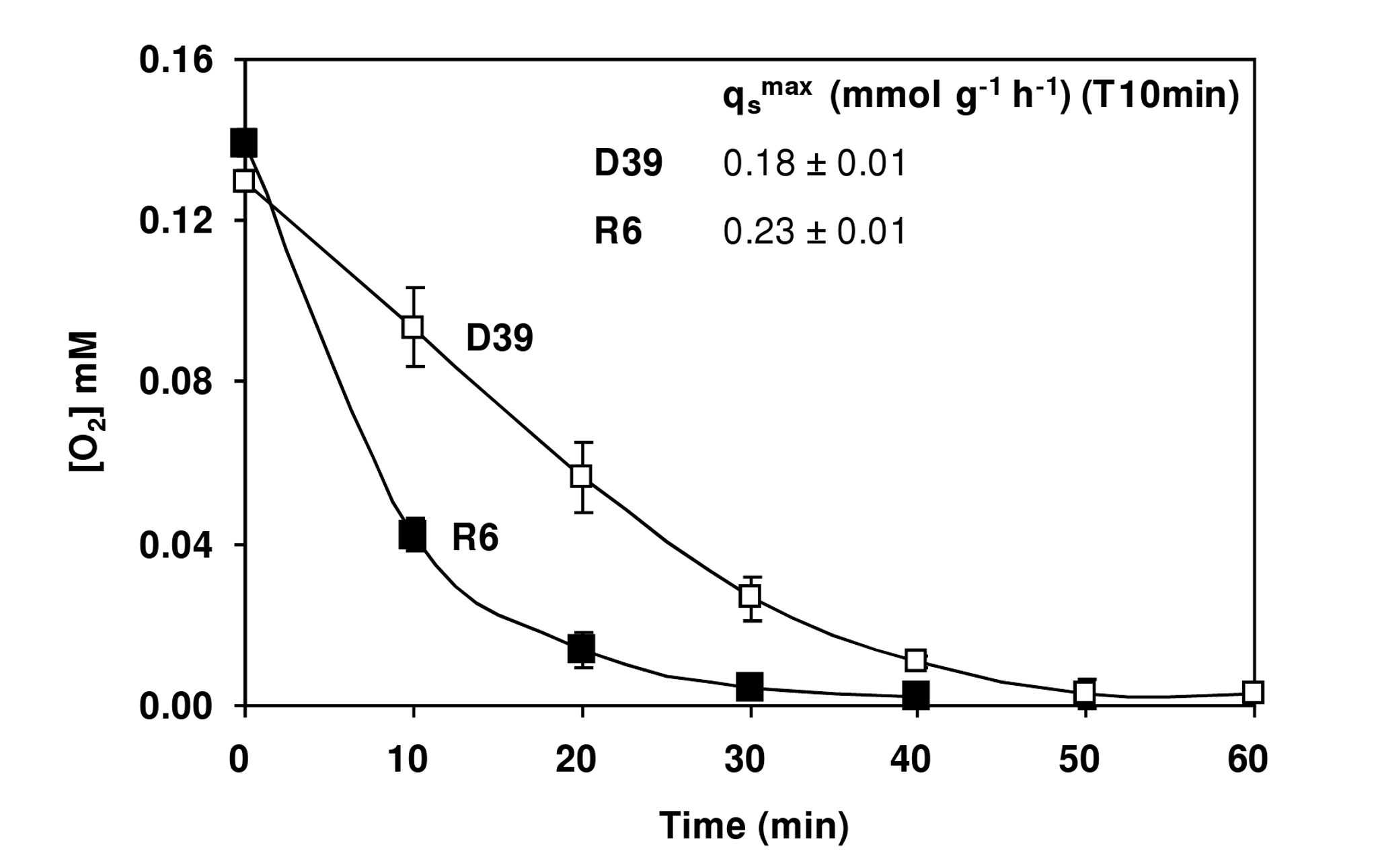

Supplement: Figure S1 — Kinetics of oxygen consumption of strains D39 and R6 grown under semi-aerobic conditions. Strains D39 (□) and R6 (▪) were grown under semi-aerobic conditions as in Fig. 2A. The oxygen consumption rates (qs max) are also shown. The plotted curves are averages of two independent experiments ± SD. (TIFF) [file pone.0058492.s001.tiff]

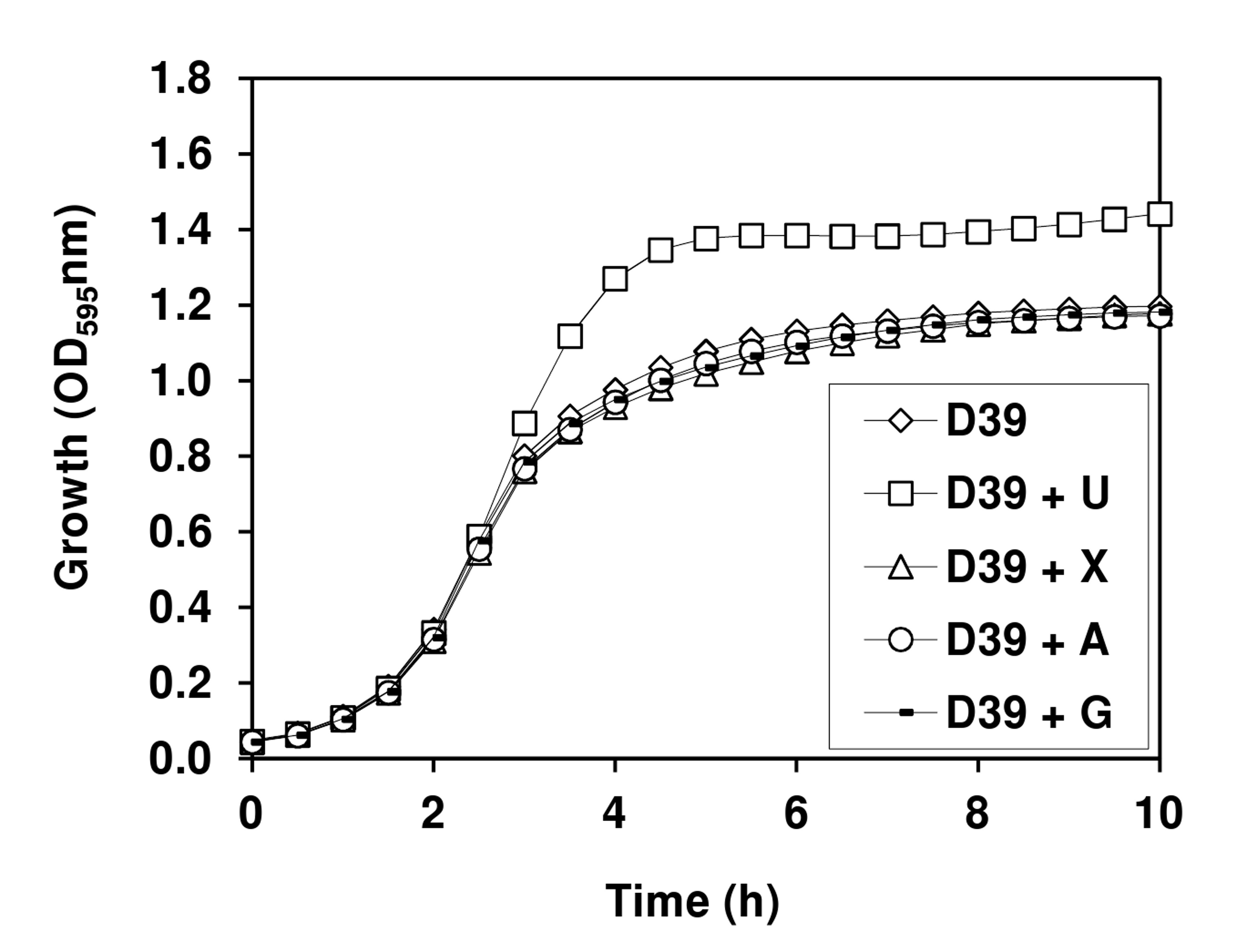

Supplement: Figure S2 — Effect on growth of increasing a single nucleobase. Growth profile of strain D39 in CDM containing 0.25% (wt/vol) glucose with 30 mg l−1 of the specified nucleobase. Cultures were prepared in 250 µl in 96-well microtiter plates and growth monitored at 595 nm and 37°C. Symbols: (⋄), G, A, X, U 10 mg l−1 each; (□), G, A, X 10 mg l−1 each plus 30 mg l−1 U; (▵), G, A, U 10 mg l−1 each plus 30 mg l−1 X; (○), G, X, U 10 mg l−1 each plus 30 mg l−1 A; (- - -), A, X, U 10 mg l−1 each plus 30 mg l−1 G. G = guanine; A = Adenine; X = Xanthine; U = Uracil. (TIF) [file pone.0058492.s002.tif]

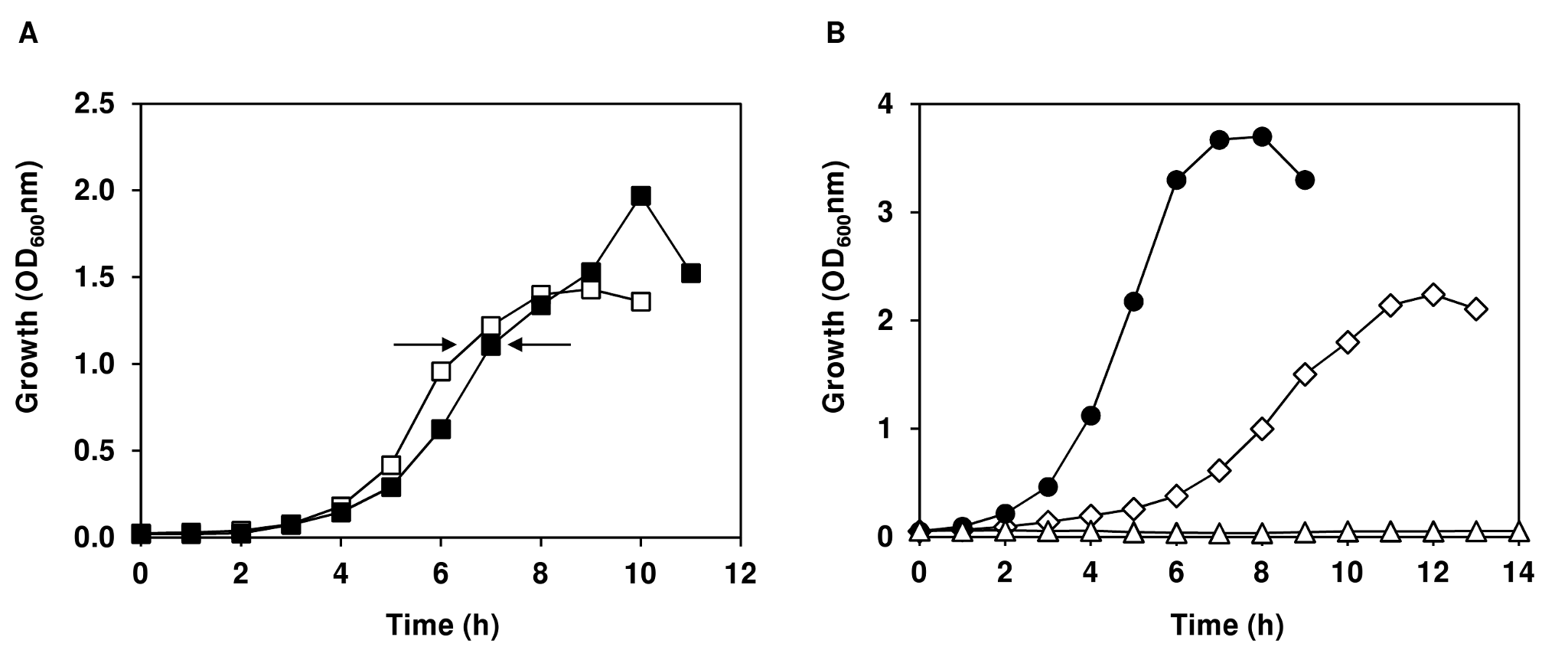

Supplement: Figure S3 — Growth profiles of D39 and R6 precultures and R6 cultures started with precultures of different ages. (A) Growth of precultures of strains D39 (□) and R6 (▪) in CDM containing 60 mM glucose, without pH control (initial pH of 6.5), at 37°C, under semi-aerobic conditions (B) Growth of strain R6 in CDM containing 60 mM glucose, under controlled conditions of pH (6.5), temperature (37°C) and atmosphere (anaerobiosis), in a 2-l bioreactor. Symbols: (•), inoculation with a preculture in late-exponential phase (LExp, 6–7 hours of incubation at 37°C, OD600 = 0.8–1.0); (⋄), inoculation with a preculture in early-stationary phase (EStat, 8–9 hours of incubation at 37°C, OD600 = 1.4–1.6); (▵), inoculation with a preculture in late-stationary phase (LStat, 18 hours of incubation at 37°C, OD600∼1). (TIF) [file pone.0058492.s003.tif]

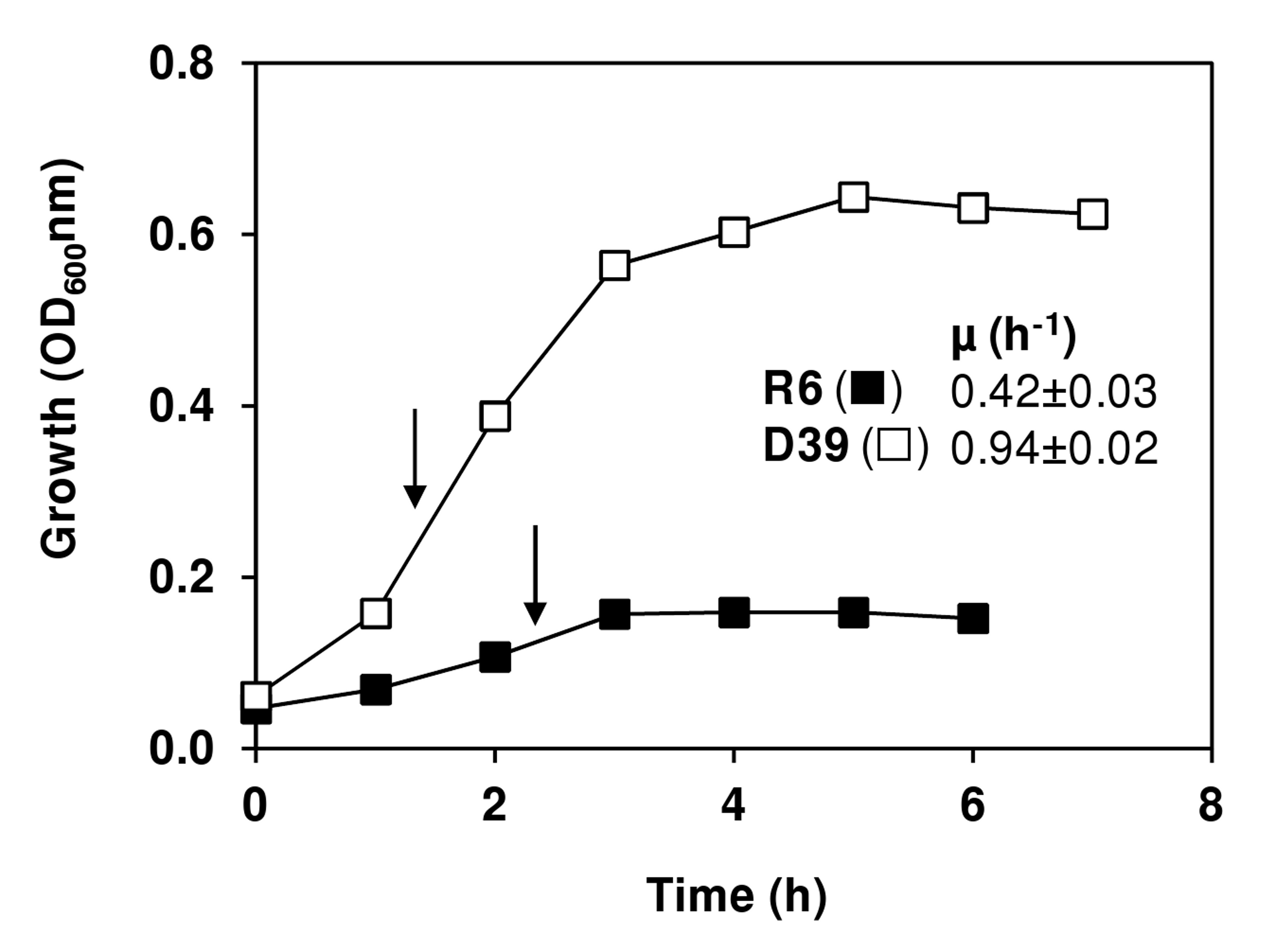

Supplement: Figure S4 — Growth profiles of cultures of strains D39 and R6 without pH control under aerobic conditions. Growth of strains D39 (□) and R6 (▪) in CDM containing 60 mM glucose, without pH control (initial pH of 6.5), at 37°C, under aerobic conditions. The arrows indicate the time-points at which cells were harvested for measurement of NADH oxidase activities. The growth rate for each culture is also indicated and the values are averages ± SD. (TIF) [file pone.0058492.s004.tif]

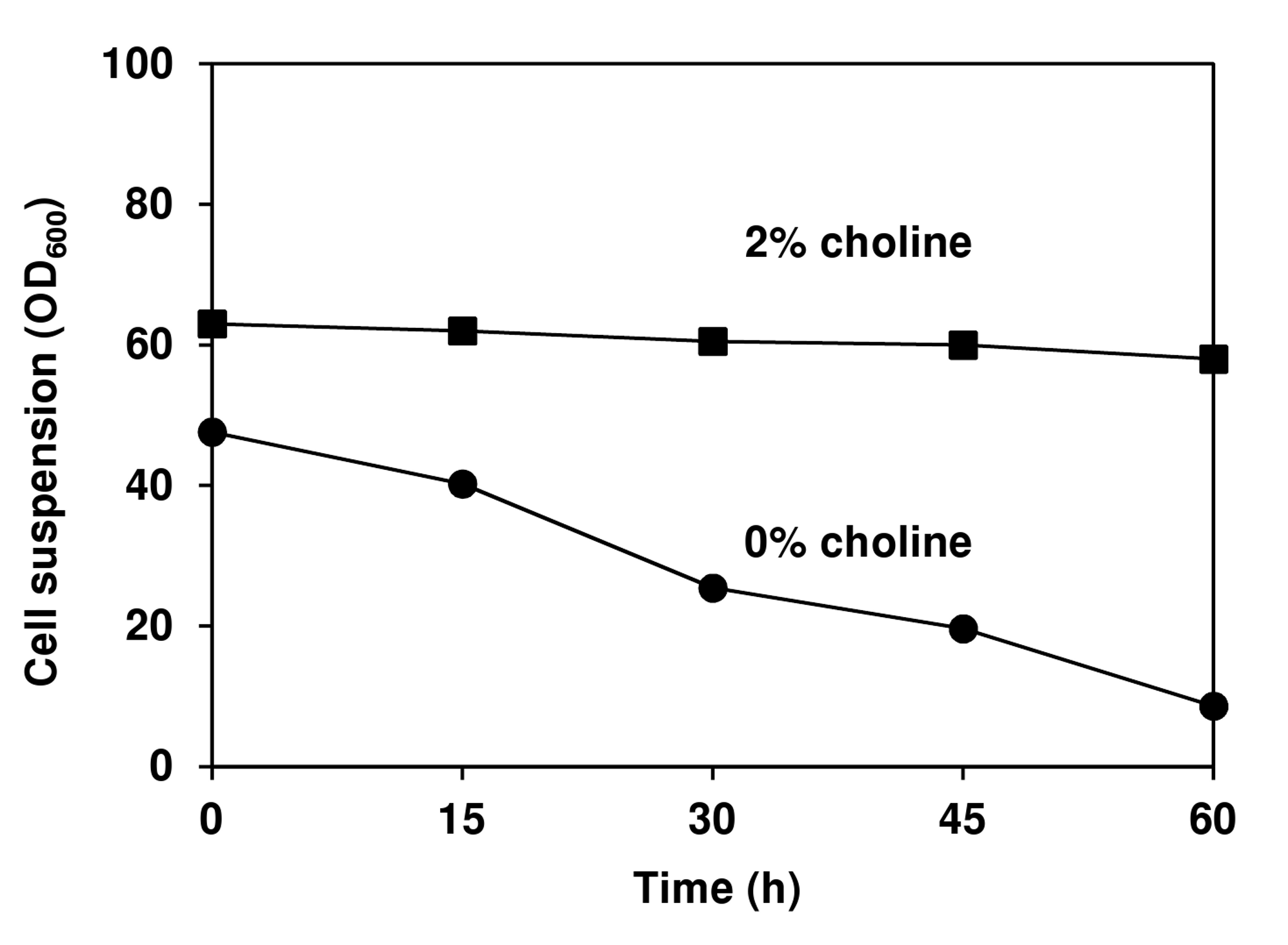

Supplement: Figure S5 — Pneumococcal lysis in resting cell suspensions. Optical density variation during glucose (20 mM) metabolism of resting cells of strain R6, grown as for in vivo NMR, suspended in 50 mM KPi with (▪) 2% or (•) 0% (wt/vol) choline. (TIF) [file pone.0058492.s005.tif]
